# KIJANI App to Promote Physical Activity in Children and Adolescents: Protocol for a Mixed Method Evaluation

**DOI:** 10.2196/55156

**Published:** 2024-05-03

**Authors:** Laura Willinger, Birgit Böhm, Florian Schweizer, Lara Marie Reimer, Stephan Jonas, Daniel A Scheller, Renate Oberhoffer-Fritz, Jan Müller

**Affiliations:** 1 Chair of Preventive Pediatrics Technical University of Munich Munich Germany; 2 Department of Informatics Technical University of Munich Munich Germany; 3 Department of Digital Medicine University Hospital Bonn Bonn Germany; 4 Associate Professorship of Didactics in Sport and Health Technical University of Munich Munich Germany

**Keywords:** physical activity, health promotion, digital health, gamification, childhood, adolescence, adolescents, adolescent, children, augmented reality, KIJANI intervention, KIJANI, intervention, user experience

## Abstract

**Background:**

The prevalence of physical inactivity among children and adolescents is alarmingly high despite the well-documented and comprehensive benefits of regular physical activity (PA). Therefore, PA promotion should start early in childhood and adolescence. Although reducing recreational screen time in children and adolescents is an urgent concern, digital approaches have the potential to make activity promotion attractive and age appropriate for the target group. KIJANI is a mobile app approach to promote PA in children and adolescents via gamification and augmented reality.

**Objective:**

This study protocol aims to describe the KIJANI intervention in detail, as well as the evaluation approach.

**Methods:**

KIJANI is based on the concept that virtual coins can be earned through PA, for example, in the form of a collected step count. With these coins, in turn, blocks can be bought, which can be used to create virtual buildings and integrate them into the player’s real-world environment via augmented reality. PA of users is detected via accelerometers integrated into the smartphones. KIJANI can be played at predefined play locations that were comprehensively identified as safe, child-friendly, and attractive for PA by the target group in a partner project. The evaluation process will be divided into 2 different stages. The phase-I evaluation will be a mixed methods approach with one-on-one semistructured interviews and questionnaires to evaluate the user experience and receive feedback from the target group. After the implementation of results and feedback from the target group, the phase-II evaluation will proceed in the form of a 2-arm randomized controlled trial, in which the effectiveness of KIJANI will be assessed via objectively measured PA as well as questionnaires.

**Results:**

The study received ethical approval from the ethical board of the Technical University of Munich. Participants for the phase-I evaluation are currently being recruited.

**Conclusions:**

The study will help to determine the efficacy, applicability, and user experience of a gamified activity promotion application in children and adolescents. Overall, digital health approaches provide easy and wide reachability at low cost and are age appropriate and attractive for the target group of adolescents. Strategies have to be developed to apply digital health approaches in the best possible way for activity promotion.

**International Registered Report Identifier (IRRID):**

DERR1-10.2196/55156

## Introduction

Physical inactivity has emerged as a worldwide pandemic, constituting the fourth leading cause of mortality worldwide [1]. A study involving 1.6 million healthy adolescents aged 11 to 17 years revealed that a staggering 81% of healthy youth are not sufficiently physically active [2]. The situation has been further exacerbated by the emergence of the COVID-19 pandemic and its associated restrictions that have further increased physical inactivity among adolescents. According to current analyses, activity levels among healthy youth decreased by 20% during the COVID-19 pandemic [3]. The prevalence of physical inactivity among children and adolescents is alarmingly high despite the well-documented and comprehensive benefits of regular physical activity (PA) including improvements in physical fitness, cardiometabolic health, bone health, cognitive performance, and mental health, among other benefits [4].

Adolescence marks a critical transitional phase during which health habits such as an active lifestyle and future health behaviors are established and manifest [5]. This implies the presence of a tracking effect of PA, whereby the more active a person is during childhood and adolescence, the more likely they are to remain active in adulthood [6,7]. Therefore, PA promotion starting from childhood and adolescence is of immense importance.

Although reducing recreational screen time in children and adolescents is an urgent concern and is strongly recommended by the World Health Organization, the potential of digital technology to make prevention and health promotion attractive and age appropriate for adolescents needs to be recognized [4]. Adolescents are power users of technology, and digital approaches allow them to reach adolescents in their personal environment. To promote PA in the particularly important phase of adolescence, we have developed a digital intervention approach called “KIJANI” that provides new opportunities for promoting and supporting PA. Specifically by targeting children and adolescents, the interdisciplinary project aims to motivate the target group through an app with a game-based concept and augmented reality to engage in PA, especially outdoors [8]. Various studies have demonstrated that gamification in health applications positively influences the behavior of the target audience [8-10] and enables children and adolescents to achieve recommended activity goals in a playful context [11]. Based on a review exploring the potential of augmented reality games for children and adolescents, the fusion of virtual and real-world elements offers promising opportunities to enhance PA and social interaction [12]. Augmented reality integrated into exergames has shown particular appeal among children [13], possibly due to their innate inclination toward play, making children an ideal target audience for gamified activity promotion [9].

This paper describes the KIJANI intervention in detail as well as the study protocol for its evaluation.

## Methods

### KIJANI Intervention

**Figure 1:**
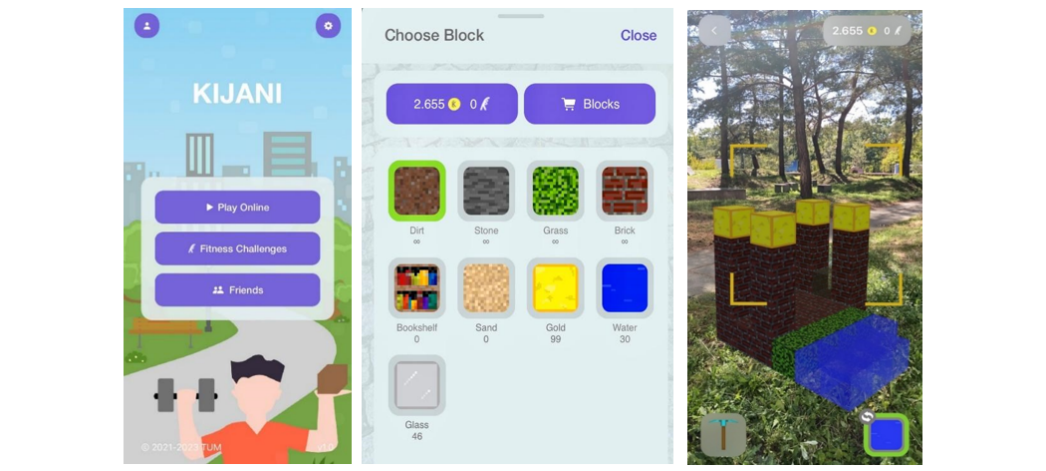
An overview of screens in the KIJANI app: KIJANI home screen (left), KIJANI shop to buy blocks (middle), and KIJANI game in augmented reality (right).

The name KIJANI is Swahili and translates to “green.” It stands as a German abbreviation for “Children & Youth: active, nature-conscious, innovative!” KIJANI is a smartphone app explicitly developed for children and adolescents to increase PA through a gamified approach. The basic game concept is that virtual coins can be “earned” through PA, for example, in the form of a collected step count. These coins can in turn be used to “buy” blocks, which can be used to create virtual buildings and integrate them into the player’s real-world environment via augmented reality, see Figure 1. Using challenge-achievement-reward loops as provided via the coin reward system in KIJANI was shown to increase the desire to play in previous research [14]. PA of users is detected via accelerometers integrated into smartphones and is linked to the KIJANI app on a technical basis.

Earning coins is based on a fitness challenge in the app, for example, consisting of 10,000 steps per day. The achievement of the main goal as well as intermediate goals are rewarded with coins. The main goal of 10,000 steps is rewarded with 50 coins, and intermediate goals, for example, 5000 steps, are rewarded with 20 coins. The blocks differ in price, for example, grass and brick cost 2 coins, water and bookshelf cost 5 coins, and the most expensive is glass and gold with 10 coins. Some blocks such as dirt and stone are free and unlimited to ensure enjoyment for the users. A progress bar in the app shows the individual progress over the course of the day, encouraging participants to reach the daily goal.

**Figure 2:**
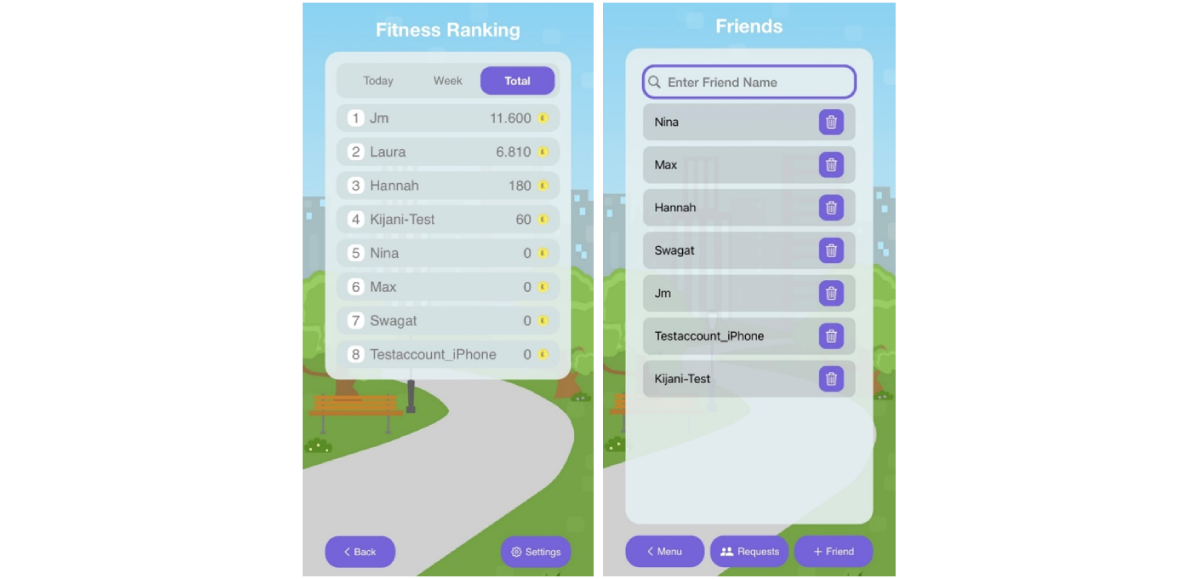
The features of the KIJANI app to connect with friends: friends ranking (left) and friends list (right).

KIJANI is designed to be played both alone and with friends in a group. To play together with friends, requests must be sent and confirmed. Then KIJANI can be played together in a group and buildings can be created together. Players can individually specify who can visit, build, and administrate buildings, which ensures that no uninvited players can participate and therefore is another safety issue in KIJANI. In addition, the individual activity behavior can be compared with friends in a ranking list, which is another incentive for PA, see Figure 2. The ranking is also available for nonfriends, as there is a setting to show all players.

**Figure 3:**
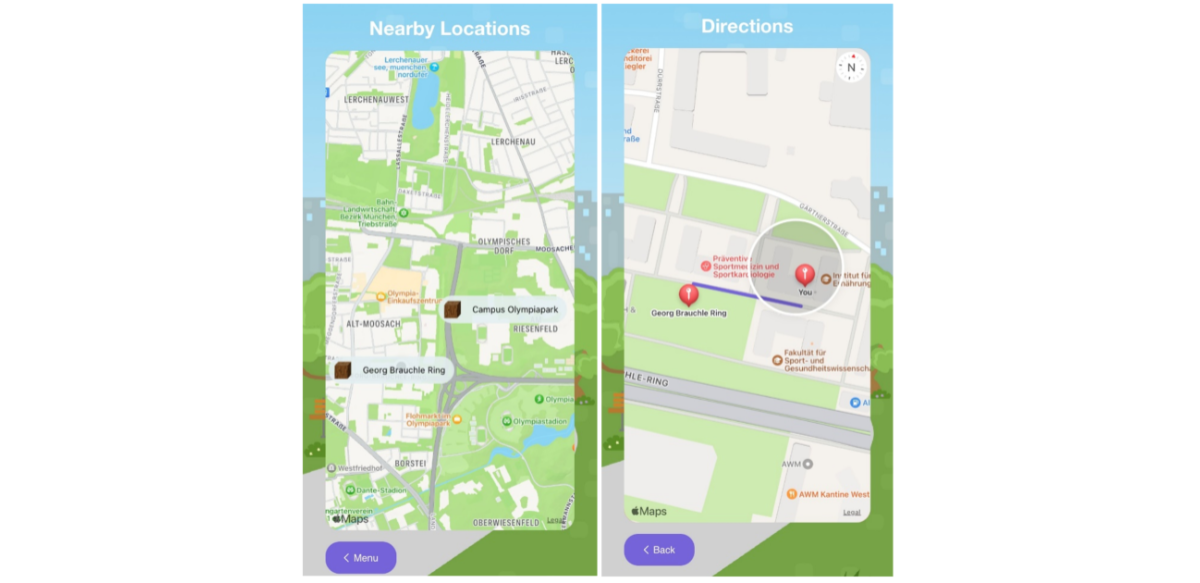
The WALKI-MUC map with play locations (left) and the navigation to play locations (right).

KIJANI was developed in cooperation with the research project “WALKI-MUC – Evaluation of physical activity opportunities and walkability perceived by children and youth in Munich,” conducted by the Associate Professorship of Didactics in Sport and Health at the Technical University of Munich. In WALKI-MUC, children and adolescents aged 6 to 17 years (n=93) identified walkable places in Munich (places within walking distance from their homes) that they considered supportive of PA. The participants provided qualitative descriptions of these PA-friendly places through participatory methods such as photovoice, walking interviews, and mapping exercises while researchers tracked the walked routes and GPS locations. Focus group discussions and the one-on-one sharing of the individual significance of these places in interviews provided insights into factors contributing to safety and attractiveness (eg, accessibility and presence of people) at the locations, as perceived by the children and adolescents. The descriptions and locations of these identified PA-friendly places of WALKI-MUC are incorporated into the KIJANI app. KIJANI can only be played in these comprehensively defined, child-friendly locations, ensuring that KIJANI can only be used in a safe and appealing environment for the target group. There is a location search feature implemented in the KIJANI app to find the play locations in the close environment. In addition, the route to the locations is displayed on the map (see Figure 3). This not only encourages users to be active but also supports a healthy lifestyle as extra steps can be accumulated.

At these play locations, a server is created in which KIJANI is played. Multiple servers can be created at 1 play location so that different buildings with different friends can be created at 1 play location. If players want to play in groups, friends have to be invited to the server to ensure that the players are in a safe play environment where no one else can intrude.

### Evaluation Design

**Figure 4:**
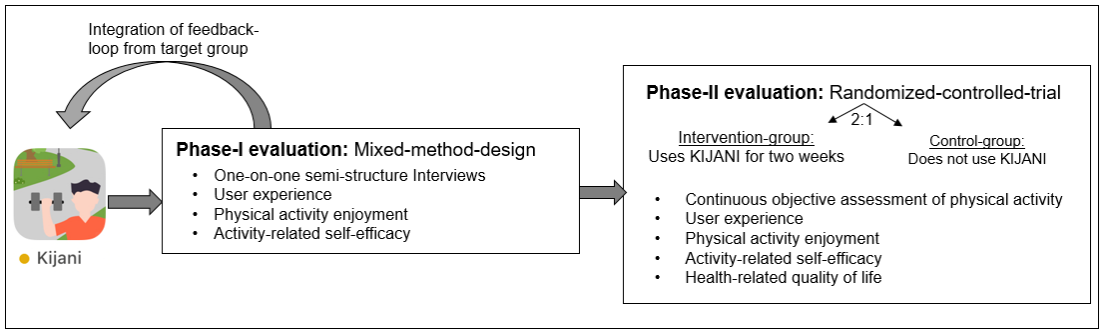
The evaluation procedure with the phase-I and phase-II evaluations and feedback loop.

It is planned to evaluate the KIJANI app in a 2-phase approach as displayed in Figure 4. The phase-I evaluation will be a mixed methods design, in which the target group evaluates the user experience of the KIJANI app. The phase-II evaluation will be a 2-arm randomized controlled trial (RCT), in which the intervention group (IG) will use the KIJANI app, while the control group (CG) will not use the KIJANI app. Within this second evaluation stage, the effectiveness of KIJANI will be assessed via objectively assessed PA as well as standardized questionnaires.

### Ethical Considerations

The study protocol has already been approved by the ethical board of the Technical University of Munich (project 2023-185-S-NP). All children and their guardians will provide written informed consent.

For the described evaluation study of KIJANI, a development version of the app is used and distributed through TestFlight. Participants are required to log into the app using pseudonyms to safeguard their data privacy. Throughout app use, step count and GPS location are shared and digitally stored in a firebase. Personal data is processed in accordance with the provisions of the current data protection laws pursuant to Art. 12 (1) BayDSG. Prior to the official release of KIJANI, all necessary international regulations will be considered.

### Participants and Enrollment

Children and adolescents at the age of 10-16 years will be included in this study. Children with walking impairments and cognitive impairments that hinder their ability to understand the task will be excluded from the study.

### Phase-I Evaluation

#### Overview

The primary outcome of the phase-I evaluation is the individual evaluation and feedback on KIJANI by the target population. Study participants will use the KIJANI app for 25 minutes in a group of 3 participants at a KIJANI play location under the supervision of the researcher. For this evaluation design, participants will have enough coins available as the main focus is on the user experience with KIJANI. Researchers ensure that all participants of a group have sufficient time and experience with the app allowing them to give informed feedback at a later stage. Afterward, the app will be evaluated qualitatively with each participant in one-on-one semistructured interviews as well as quantitatively with standardized questionnaires on user experience, PA enjoyment, as well as activity-related self-efficacy.

#### Qualitative Evaluation

**Figure 5:**
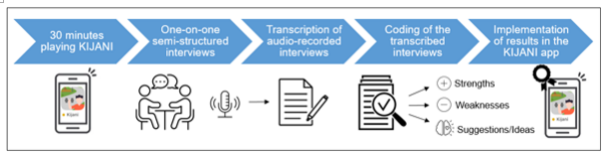
The procedure of the qualitative evaluation process of KIJANI.

In one-on-one semistructured interviews, participants will report on their opinions and experiences with KIJANI. For this purpose, a previously generated interview guide, including mainly open‐ended questions, will be used. The interview guide includes warm-up questions to establish a good relationship with the participants and to inquire about their attitudes toward smartphone use, digital games, as well as PA. The main part of the interview is related to the participants’ opinions on the KIJANI app, including questions about what participants liked and what they would improve in the app. At the end of the interview, participants will have another opportunity to express further comments and ideas. All interviews will be conducted in person, audio recorded, and then transcribed verbatim. Interviewers will not be familiar with the participants prior to study initiation. The interviews will be evaluated by the respective interviewer. For evaluation purposes, categories will be generated guided by a systematic reduction process during data analysis. Text sequences will be classified into suitable categories, and thereby codes will be generated inductively. Possible uncertainties concerning the coding scheme will be discussed by the researchers and homogeneously adjusted. Data collection will proceed until data saturation has been achieved. Data will be considered saturated when recurring codes are observed across various interviews, accompanied by a decreased emergence of new codes during analysis (inductive thematic saturation). The qualitative evaluation process is displayed in Figure 5.

#### User Experience

In addition, the user experience with the KIJANI app is evaluated with a user experience questionnaire [15]. User experience thereby summarizes different aspects that are important for the subjective evaluation of a product: usability aspects, joy of use, as well as aesthetic design. The user experience questionnaire consists of 26 bipolar terms (pairs of opposites, eg, annoying and enjoyable), which are evaluated over 7 levels. The items consist of the following 6 domains: effectiveness, transparency, predictability, stimulation, originality, and attractiveness. The questionnaire shows high reliability and construct validity [16].

#### PA Enjoyment

PA enjoyment is evaluated using the short version of the PA Enjoyment Scale (PACES-S). The PACES-S is a measurement tool commonly used in intervention research to measure enjoyment of PA, as it is closely associated with adherence and compliance to PA guidelines [17]. The German version of the PACES is reliable and valid for use in children and adolescents [18]. The short version of the questionnaire consists of 4 items with 5 scales (eg, PA brings me joy). It shows comparable measurement properties to the long version of the PACES and is therefore used in this study [19].

#### Activity-Related Self-Efficacy

Perceived self-efficacy in the context of PA is measured using the German version of the PA Self-Efficacy Scale. The scale consists of 8 items, each of which is answered on a 5-point Likert scale ranging from 1 (“do not agree at all”) to 5 (“fully agree”). The self-efficacy scale for PA measures activity-related self-efficacy with 6 items and activity-related social support from family and friends via the remaining 2 items. The reliability and validity of the German version were shown in a study with 454 schoolchildren [20].

### Phase-II Evaluation

#### Overview

After the implementation of the results of the first evaluation phase, the second evaluation in the form of an RCT will be conducted. The IG will use the KIJANI app in their everyday life over a period of 2 weeks, while the CG sticks to their normal everyday life without using the KIJANI app. Randomization will be 2:1 into IG and CG, with the randomization process clustered by age and gender to ensure equal distribution in the groups. The phase-II evaluation sample size was calculated with G*power (2-sided α of 0.05, power 0.9). A sample of 62 participants is planned for this study. Of these, 41 participants will be assigned to the IG and 21 participants will be assigned to the CG. After completion of the study, the CG will also have the opportunity to use the KIJANI app.

The primary outcome of the phase-II evaluation is the everyday PA of the children and adolescents. PA is objectively recorded in the form of daily step count via accelerometers integrated into the smartphone over the entire study period in IG and CG. Through the KIJANI intervention, we hope to increase the PA of children and adolescents by 10%.

Secondary end points are health-related quality of life, activity-related self-efficacy, PA enjoyment, as well as the user experience with the app. The last 3 will be investigated as described above in the phase-I evaluation. Secondary end points will be assessed before and after the intervention period.

#### Health-Related Quality of Life

Health-related quality of life is self-assessed by the Kinder Lebensqualitätsfragebogen (KINDL) questionnaire. The KINDL is a generic instrument for assessing health-related quality of life in children and adolescents. For study participants younger than 14 years, the KINDL child version is used, and for study participants 14 years and older, the KINDL adolescent version is used. The KINDL is a short, methodologically tested, and flexible measurement instrument consisting of 24 questions on the 6 domains of body/physis, feelings/psychology, self-assessment, family, friends, and school [21].

### Statistical Methods

The data analysis will involve both descriptive and analytical analyses. Initially, descriptive analysis will focus on the sociodemographic characteristics of the study population. Normal distribution of the data will be tested with the Shapiro-Wilk test. In terms of dropouts, intention-to-treat analysis will be performed using multiple imputations, to be able to calculate with a complete data set. To capture the longitudinal development of the activity level throughout the study period, a linear mixed model will be used to compare the change of the PA behavior over time between IG and CG. To investigate differences between the groups in activity-related self-efficacy, health-related quality of life, and PA enjoyment, these will be compared with 2-tailed *t* test or Mann-Whitney *U* test as appropriate depending on the distribution of the data. All analyses will be performed with RStudio (version 4.1.2; RStudio Team), with the level of significance set to 2-sided *P* values <.05 for all tests. RStudio was used for its tailored integration with the R programming language, providing an efficient and organized environment for reproducible data analysis, visualization, and documentation in our scientific study.

## Results

Once the phase-I evaluation is completed, the outcomes will be discussed with the app development team and adjustments will be incorporated in KIJANI through this feedback loop. Data collection for the phase-I evaluation started in August 2023 and recruitment is currently still ongoing.

## Discussion

### Principal Findings

KIJANI is a mobile game explicitly developed for PA promotion in children and adolescents with a special focus on creativity as well as PA in the outdoor setting. The objective of the KIJANI intervention is to increase everyday PA in adolescents through a digital intervention approach using gamification and augmented reality. This study protocol describes the KIJANI intervention in detail, as well as the evaluation approach.

Even though activity promotion is highly indicated in adolescents, this young target group is difficult to reach, and adherence to intervention approaches, especially over the long term, is mostly rather low [22].

Several previous studies have assessed the effectiveness of mobile health approaches to increase PA in healthy adolescents. A systematic review on the effects of mobile health to increase PA concluded that mobile devices and apps may be an effective strategy for promoting PA in adolescents as they have high technology use. However, overall, there is only a small number of studies; a lack of RCTs; and a high risk of bias in this research sector, mostly including self-reported measures with the risk for recall as well as report bias [23]. Another systematic review on the quality and features of apps to improve PA, sedentary behavior, and diet also reported that generally, very few apps are available that specifically target children and adolescents and app quality was only moderate [24]. Overall, there is a need for high-quality app-based activity promotion approaches especially in children and adolescents.

Some previous studies have assessed the effects of digital gamification approaches on activity promotion. A meta-analysis revealed that the popular mobile augmented reality game “Pokémon Go” was associated with a significant increase in the users’ daily step count. In the game, participants are required to travel to different locations to capture virtual characters. Even though the game was not primarily designed to promote PA, it has reached masses of users and has shown to have the potential to influence their health behavior at least in the short term [25]. Long-term results are rare, but it seems that the novelty effect tends to wear off with time, and after 3 months, 80% of players stopped using the app [26].

Direito et al [27] assessed the effect of 2 smartphone apps, 1 immersive and 1 nonimmersive app, on cardiorespiratory fitness and PA levels in healthy young people. Both apps consisted of 8-week training programs, whereby in the immersive app the training program was embedded with a story where the user has to collect supplies and protect a town from zombies. After 8 weeks, they were not able to report any intervention effect compared with a CG in both apps. Adherence to app use was a major concern during their investigation [27].

Garde et al [28] evaluated another smartphone app called “MobileKids Monster Manor,” in which behavioral psychology, positive peer pressure, and rewards are used with a monster character theme to increase PA in healthy adolescents. In a school-based environment, this intervention increased PA over a short-term period [28].

### Limitations

Several limitations should be considered when discussing this study protocol. In the phase-I evaluation, one-on-one interviews carry the risk of social desirability bias, where participants may provide responses perceived as socially acceptable rather than expressing their true opinions or behaviors. The phase-II evaluation carries the risk of the Hawthorn effect, which implies the awareness of being observed and studied that impacts people’s behavior. Based on this effect, PA levels might be higher than normal throughout the measurement period of 2 weeks.

### Conclusions

Overall, digital health approaches provide easy and wide reachability at low cost and are age appropriate and attractive for the target group of adolescents. Strategies have to be developed to apply digital health approaches in the best possible way for activity promotion. The study will help to determine the efficacy, applicability, and user experience of a gamified activity promotion application in children and adolescents.

## Abbreviations

CG: control group
IG: intervention group
KINDL: Kinder Lebensqualitätsfragebogen
PA: physical activity
PACES: Physical Activity Enjoyment Scale
PACES-S: short version of the Physical Activity Enjoyment Scale
RCT: randomized controlled trial

## Data Availability

The data sets generated and analyzed during this study are available from the corresponding author on reasonable request.
